# Lower expressions of the human bitter taste receptor TAS2R in smokers: reverse transcriptase-polymerase chain reaction analysis

**DOI:** 10.1186/1617-9625-12-12

**Published:** 2014-08-15

**Authors:** Mieko Aoki, Tetsuya Takao, Kyoichi Takao, Fumihiko Koike, Narufumi Suganuma

**Affiliations:** 1 Department of food and nutrition, Sanyo Gakuen College, 1-14-1 Hirai, Naka-ward, Okayama 703-8501, Japan; 2 Department of nutrition and health promotion, Showa Women’s University, Tokyo, Japan; 3 School of Medicine, Nihon University, Tokyo, Japan; 4 Department of Dentistry and Oral Surgery, Nihon University, Tokyo, Japan; 5 Department of Medicine, Kochi University, Kochi, Japan

**Keywords:** Cigarette, Human, TAS2R, RT-PCR

## Abstract

**Background:**

Despite the fact that smokers have deficit in detecting taste, particularly bitter taste, no study has investigated its biological correlate.

**Methods:**

In this context, we compared the expression of the bitter taste receptor gene, taste 2 receptor (TAS2R) in the tongues of smokers and non-smokers. Tissue samples were collected from the lateral portion of the tongues of 22 smokers and 22 age- and gender-matched healthy volunteers (19 males and three females) with no history of smoking. Reverse transcriptase-polymerase chain reaction was used to examine the expression of TAS2R in the two groups, and the effect of aging on TAS2R expression was also assessed.

**Results:**

TAS2R expression was significantly lower among smokers than non-smokers (*t* = 6.525, *P* < .0001, 11.36 ± 6.0 vs. 2.09 ± 2.8, mean ± SD, non-smokers vs. smokers). Further, a positive correlation between age and expression of TAS2R was observed in non-smokers (*r* = .642, *P* = .001), but not smokers (*r* = .124, *P* = .584). This correlation difference was significant (*Z* = 1.96, *P* = .0496).

**Conclusions:**

Smokers showed a significantly lower expression of the bitter taste receptor gene than non-smokers, which is potentially caused by their inability to acquire such receptors with age because of cigarette smoking, in contrast to non-smokers.

## Background

Accumulating evidence has demonstrated that cigarette smoking induces non-critical adverse effects [1-5] in addition to the well-established critical adverse effects such as the development of cancers and coronary artery diseases [6,7]. Among the non-critical adverse effects of smoking, bitter taste impairment is particularly important in the field of public health [8-11], not only because it affects the quality of life of smokers but also because it may promote the continuation of smoking which will eventually cause critical adverse effects in both smokers and their relatives. However, despite the importance of this as a potential mechanism for the continuation of smoking [12-14], to the best of our knowledge no study has investigated the biological mechanism of this bitter taste disability.

Bitter tastes are perceived by taste receptor cells (TRC) which are assembled into taste buds [15]. In humans, TRCs detect bitter stimuli via a bitter taste receptor, the taste 2 receptor (TAS2R) G-protein-coupled receptor (GPCR) [15]. Although their roles have yet to be elucidated, human genome studies have shown that the TRC-expressed GPCR multi-gene family consists of 25 T2R members [16]. They are named by adding numbers at the end, such as TAS2R1 or TAS2R3.

There are some studies that suggested potential genetic effect of smoking cigarette, such as association between smoking cigarettes and SNP expression [17]. We hypothesized that cigarette smoking induces a lower expression of TAS2R in smokers compared with non-smokers. To test this, we performed reverse transcriptase-polymerase chain reaction (RT-PCR) analysis of TAS2R expression in tongue tissue samples of both groups.

## Methods

### Participants and clinical evaluation

Twenty-two smokers (20 males and two females) with no history of taste disorders were recruited to participate in the study. They are mainly college students and acquaintances of the authors. The following information was obtained from each of these participants: age (years), smoking duration (years), and the number of cigarettes smoked per day; these data were also used to determine the Brinkman index, which is defined as the number of cigarettes smoked daily multiplied by the number of smoking years. Twenty-two age- and gender-matched healthy volunteers (19 males and three females) with no history of smoking were employed as controls. To avoid age-associated loss of expression, we excluded participants aged 65 years or over from both groups.

The ethics committee of Sanyo Gakuen College, Showa women’s University and Medical school of Nihon University approved the current study. After a complete explanation of the study to the subjects, written informed consent was obtained from every participant.

### Sample collection

We adopted a scraping method to collect tissue samples for this study because it was difficult to perform an excisional biopsy in several cases. This scraping method is so minimally invasive that it is often used to collect tissue samples from the oral cavity. We considered it suitable as a tissue-sampling method in the present study for the examination of taste receptor gene expression of the tongue. Thus, by scraping with plastic tube, we obtain the epithelium specimen from tip, dorsum and foliate papilla in the tongue. None of the participants had anything to eat or drink for at least 90 min before scraping the tongue surface. The specimens were vortexed with TRIzol Reagent (Invitrogen) of 0.5ml for 30 seconds. Then, upper aqueous phase was transferred carefully into fresh tube without disturbing the interphase and used as specimens of interest. The lower red phenol-chloroform phase was used for assessment of potential contamination of DNA. Specimens were refrigerated at temperature of -5°C for a night then -35°C for a few days before RT-PCR. Based on the instruction of TRIzol Reagent, all RNAs were purified from the specimen. To make sample to be suitable for RT-PCR, the pellet of total RNA was dissolved in DEPC-treated water of 10 μL. The concentration of total RNA in the sample was measured. The sample was stored at temperature of -80°C.

### Reverse transcriptase-polymerase chain reaction

RT-PCR was performed as previously described [18]. Briefly, the primer pairs (Table 1) were designed from 5’ sides and 3’ sides of TAS2R genes. Using SuperScript III (Invitrogen, Carlsbad, CA, USA) and random primers (Invitrogen, Carlsbad, CA, USA), first strand cDNAs were reverse-transcribed from 0.65μg total RNA. The cDNA of 0.5μL was used as a template for PCR reaction. The PCR was performed with ExTaq (Takara Bio Inc., Shiga, Japan). The PCR amplification was started with a denaturation step at 95°C for 5min followed by 35 cycles consisting of denaturation (95°C for 30 sec), annealing (59°C for 30 sec) and extension (72°C for 60 sec). The oligonucleotides used for TAS2R amplification is shown in Table 1. The sample was incubated for seven minutes at 72°C. After PCR reaction, the specimen was stored at 4°C. The length and amount of PCR product was analyzed using 2100 Agilent Bioanalyzer (Agilent Technology, Tokyo, Japan) with 7500 Agilent DNA Reagent.

**Table 1:** List of the sequences of the oligonucleotides used for TAS2R amplification

**TAS2R**	**Accession number**	**Primer name**	**Primer**	**Product length**
TAS2R1	NM_019599	TAS2R1/F	atg cta gag tct cac ctc att atc	900 bp
		TAS2R1/R	tca ctg aca gca ctt act gtg gag g	
TAS2R3	NM_016943	TAS2R3/F	atg atg gga ctc acc gag ggg g	951 bp
		TAS2R3/R	cta aga gaa aat ggg tcc ctt gg	
TAS2R4	NM_016944	TAS2R4/F	atg ctt cgg tta ttc tat ttc	900 bp
		TAS2R4/R	cta ttt ttt gaa aca aag aat c	
TAS2R5	NM_018980	TAS2R5/F	atg ctg agc gct ggc cta gga ctg	900 bp
		TAS2R5/R	tca tgg gcc cca gca tct ccg agc	
TAS2R7	NM_023919	TAS2R7/F	atg gca gat aaa gtg cag act ac	957 bp
		TAS2R7/R	tca gat ttg ttt atg ttg ttg ga	
TAS2R8	NM_023918	TAS2R8/F	atg ttc agt cct gca gat aac	930 bp
		TAS2R8/R	tca tat cat gca ggc aat ttt tc	
TAS2R9	NM_023917	TAS2R9/F	atg cca agt gca ata gag gc	939 bp
		TAS2R9/R	cta tgg aac aaa agg ctt tc	
TAS2R10	NM_023921	TAS2R10/F	atg cta cgt gta gtg gaa ggc	924 bp
		TAS2R10/R	cta tgt gac tct gag att ttt cc	
TAS2R13	NM_023920	TAS2R13/F	atg gaa agt gcc ctg ccg ag	912 bp
		TAS2R13/R	tca tcg ttt agc cca tac c	
TAS2R14	NM_023922	TAS2R14/F	atg ggt ggt gtc ata aag ag	954 bp
		TAS2R14/R	tca aga tga ttc tct aaa ttc	
TAS2R16	NM_016945	TAS2R16/F	atg ata ccc atc caa ctc ac	876 bp
		TAS2R16/R	cta gca ctt tcc ctt	
TAS2R19	NM_176888	TAS2R19/F	atg atg tgt ttt ctg ctc atc	900 bp
		TAS2R19/R	tca gcg tgt cat ctg cca caa a	
TAS2R20	NM176889	TAS2R20/F	atg atg agt ttt cta cac att g	930 bp
		TAS2R20/R	cta tgg agt tga ctg gtt ctg tcc	
TAS2R30	NM_001097643	TAS2R30/F	atg ata act ttt ctg ccc atc a	960 bp
		TAS2R30/R	cta gaa gac aca caa tgc ccc tc	
TAS2R31	NM176885	TAS2R31/F	atg aca act ttt ata ccc atc	930 bp
		TAS2R31/R	cta tgg aga tga agg ctt ctc tcc	
TAS2R38	NM_176817	TAS2R38/F	atg ttg act cta act cgc atc	1002 bp
		TAS2R38/R	tca gca cag tgt ccg gga atc t	
TAS2R39	NM_176881	TAS2R39/F	atg cta ggg aga tgt ttt cct cc	1017 bp
		TAS2R39/R	tca cag agt cca ctc ttt tgg gt	
TAS2R40	NM_176882	TAS2R40/F	atg gca acg gtg aac aca gat g	972 bp
		TAS2R40/R	tca cag agt ctg ccc ttt tag gt	
TAS2R42	NM_191429	TAS2R42/F	atg gcc acc gaa ttg gac	945 bp
		TAS2R42/R	cta caa agg taa agg gtt tgg tg	
TAS2R43	NM176884	TAS2R43/F	atg ata act ttt cta ccc atc	930 bp
		TAS2R43/R	cta tgg aga tga agt ctt ctc tcc	
TAS2R45	NM_176886	TAS2R45/F	atg ata act ttt ctg ccc atc	900 bp
		TAS2R45/R	tca gta cct cat ttg cca caa aac tg	

### Assessment of potential contamination of DNA

After ethanol precipitation of the lower red phenol-chloroform phase of the specimen, genomic DNA was extracted. Then, extracted genomic DNA was analyzed by agarose gel electrophoresis.

### Statistical analyses

Independent *t*-tests were performed to compare the differences in age between smokers and non-smokers, and in the expression of the bitter taste receptor gene TAS2R between smokers and non-smokers. Statistical significance was set at *P* < .05.

Independent *t*-tests were also performed to compare the difference in the expression of TAS2R subfamily genes between smokers and non-smokers. Because we had no *a priori* hypothesis for potential expression level differences in the TAS2R subfamily, statistical significance was set at *P* < .0024 (=.05/21, number of TAS2R subfamily members successfully measured), correcting multiple comparisons using Bonferroni method.

### Correlation analysis

To explore the potential effect of age on the expression of bitter taste receptor genes, we performed Pearson’s correlation analyses between age and TAS2R expression in non-smokers and smokers separately. Differences in correlations between groups were examined with the Fisher r-to-z transformation [19]. The statistical threshold was set at *P* < .05.

Further, to examine the effect of potential factors related to bitter taste receptor gene expression among smokers, we conducted Pearson’s correlation analyses between TAS2R expression and period of smoking (years), number of cigarettes smoked daily, and the Brinkman index. The statistical threshold was also set at *P* < .05.

## Results

### Demographic characteristics

There was no significant difference in the age of the participants between the smokers and non-smokers (46.0 ± 11.4 years (mean ± SD; range, 23–65) and 41.5 ± 15.8 years (mean ± SD; range, 23–65), respectively; *P* = .290) (Table 2). The mean duration of smoking was 24.3 ± 9.7 years (mean ± SD), and an average of 17.4 ± 7.0 cigarettes were smoked daily (mean ± SD). Consequently, the mean Brinkman index was 430.0 ± 246.7 (mean ± SD) in smokers (Table 2).

**Table 2:** Demographic characteristics of participants

**Variable**	**Non-smoker (n = 22)**		**Smoker (n = 22)**		***t*-test**	
	**Mean**	**SD**	**Mean**	**SD**	***t*-value**	***P*-value**
Age (years)	41.5	15.8	46.0	11.4	1.1	.290
Duration of smoking (years)	NA	NA	24.3	9.7	NA	NA
Number of cigarettes smoked daily	NA	NA	17.4	7.0	NA	NA
Brinkman index	NA	NA	430	246.7	NA	NA

Abbreviations: NA, not applicable; SD, standard deviation.

### Assessment of potential contamination of DNA

**Figure 1:**
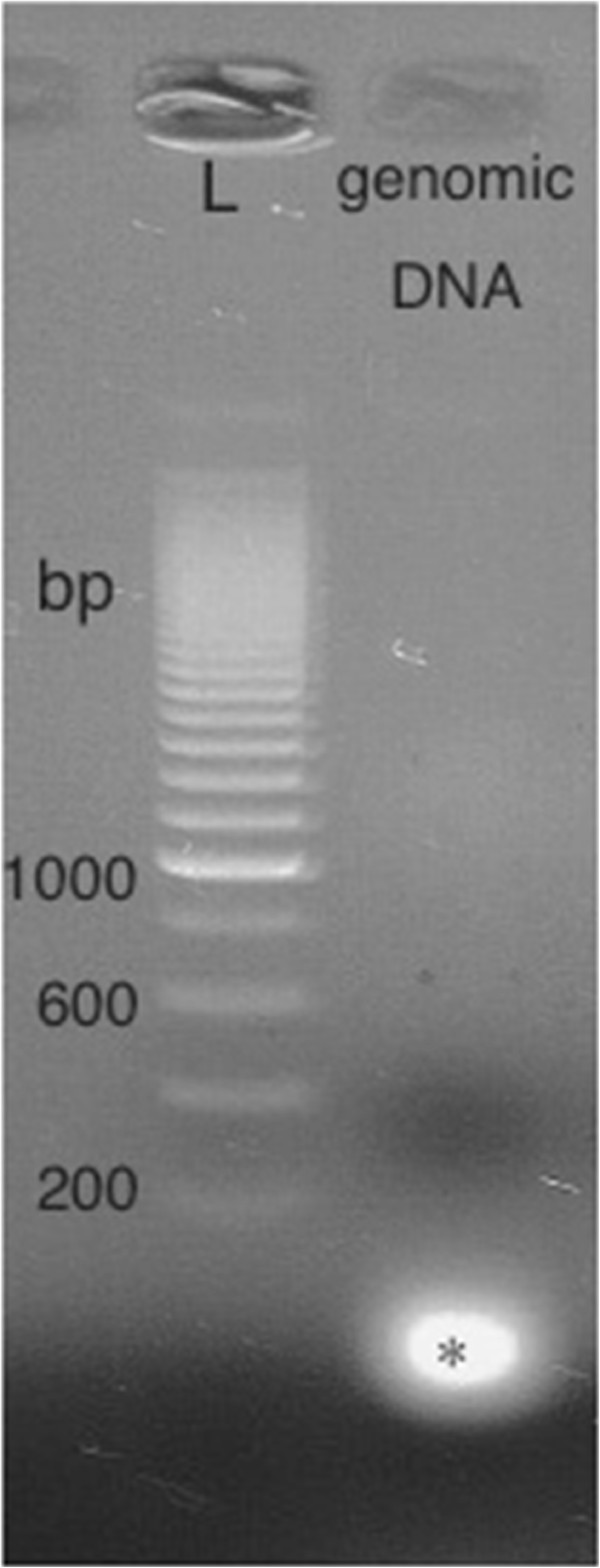
**Genomic DNA fragmentation by a homogenate.** The genomic DNA isolated from the lower phase of the TRIzol Reagent treated specimen was observed only in 200 bp or less (indicated by “*”), suggesting that genetic DNAs were fragmented into small fraction during the process of homogenate and there is no genomic DNAs that are large enough to express TAS2R in the upper phase.

As shown in Figure 1, genomic DNAs were fragmented into less than 200bp.

### Comparison of TAS2R expression between smokers and non-smokers

The mean expression of TAS2R in smokers and non-smokers was 2.09 and 11.36, respectively. This difference was statistically significant, with smokers having a significantly lower TAS2R expression compared with non-smokers (*t* = 6.525, *P* < .0001, Table 3).

**Table 3:** Mean age and expression of TAS2R and its subfamily

**Variable**	**Non-smoker (n = 22)**		**Smoker (n = 22)**		**Independent *t*-tests**		
	**Mean**	**SD**	**Mean**	**SD**	***t*-value**	**df**	***P*-value**
Total TAS2R expression	11.36	6.04	2.09	2.81	6.525	42	< .0001*
TAS2R subfamily expression							
TAS2R1	.55	.51	.18	.59	2.191	42	.0341
TAS2R3	.68	.48	.45	.67	1.295	42	.2024
TAS2R4	.32	.48	.36	.73	.25	42	.8074
TAS2R5	.36	.49	.27	.70	.50	42	.6217
TAS2R7	.64	.49	.09	.43	3.928	42	.0003*
TAS2R8	.82	.39	.55	.67	1.643	42	.1078
TAS2R9	.82	.39	.32	.57	3.391	42	.0011*
TAS2R10	.77	.43	.36	.58	2.657	42	.0111
TAS2R13	.45	.51	.18	.59	1.643	42	.1078
TAS2R14	.36	.49	.32	.57	.28	42	.7781
TAS2R16	.64	.49	.27	.46	2.542	42	.0148
TAS2R19	.59	.50	.14	.47	3.104	42	.0034
TAS2R20	.27	.46	0	0	2.806	42	.0076
TAS2R30	.73	.46	0	0	7.483	42	< .0001*
TAS2R31	.27	.46	0	0	2.806	42	.0076
TAS2R38	.59	.50	0	0	5.508	42	< .0001*
TAS2R39	.55	.51	.18	.50	2.386	42	.0216
TAS2R40	.64	.49	.36	.73	1.457	42	.1525
TAS2R42	.23	.43	0	0	2.485	42	.0170
TAS2R43	.41	.50	.14	.47	1.862	42	.0696
TAS2R45	.50	.51	0	0	4.583	42	< .0001*

*significant after Bonferroni correction.

### Comparison of TAS2R subfamily expression

Independent *t*-tests revealed that the expression of five members of the TAS2R subfamily was significantly lower among smokers compared with non-smokers. These members are: TAS2R7 (*t* = 3.928, *P* = .0003, .64 ± .49 vs. .09 ± .43, mean ± SD, non-smokers v. smokers), TAS2R9 (*t* = 3.391, *P* = .0011, .82 ± .39 vs. .32 ± .57, mean ± SD, non-smokers v. smokers), TAS2R30 (*t* = 7.483, *P* < .0001, .73 ± .46 vs. 0 ± 0, mean ± SD, non-smokers v. smokers), TAS2R38 (*t* = 5.508, *P* < .0001, .59 ± .50 vs. 0 ± 0, mean ± SD, non-smokers v. smokers), and TAS2R45 (*t* = 4.583, *P* < .0001, .50 ± .51 vs. 0 ± 0, mean ± SD, non-smokers v. smokers) (Table 2). Although it did not reach significance, the mean expression of TAS2R4 was higher among smokers (.32 ± .48, mean ± SD) than non-smokers (.36 ± .73, mean ± SD) (*P* = .108; Table 2).

### Correlation analyses

**Figure 2:**
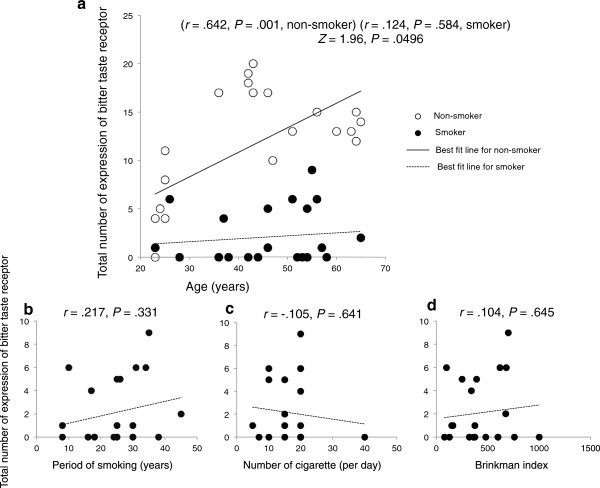
**Relation between number of expression of TAS2R and age and smoking behaviors.** A significant positive correlation was observed between age and bitter taste receptor expression in non-smokers but not in smokers **(a)**. There was no significant association between bitter taste receptor expression and duration of smoking **(b)**, the number of cigarettes smoked daily **(c)**, and the Brinkman index **(d)**.

Age was significantly positively correlated with the expression of TAS2R in the non-smoker group (*r* = .642, *P* = .001) but not in the smoker group (*r* = .124, *P* = .584) (Figure 2a). Fisher’s r-to-z transformation revealed a significant difference in the correlations between the smokers and non-smokers (*Z* = 1.96, *P* = .0496), indicating that the age–expression of bitter taste receptor relationship in smokers was significantly different from that of non-smokers (Figure 2a).

Pearson’s correlation analyses found no significant association between TAS2R expression and duration of smoking (*r* = .217, *P* = .331) (Figure 2b), number of cigarettes smoked daily (*r* = -.105, *P* = .641) (Figure 2c), or the Brinkman index (*r* = .104, *P* = .645) (Figure 2c).

## Discussion

In the current study, we detected a significantly lower expression of TAS2R in smokers compared with age- and gender-matched non-smokers. Sub-analyses also demonstrated that bitter taste receptor gene expression was significantly lower in five of the 21 receptors studied, TAS2R7, 9, 30 38, and 45. Although we did not investigate whether the current smokers have impaired taste, our results strongly suggest that the well-documented disturbance of taste seen in smokers derives from the lower-than-normal expression of TAS2R caused by smoking [8-11]. There are several potential substances that may affect the expression of TAS2R, the most potentially relevant of which is nicotine [20]. Indeed, previous studies have reported that nicotine decreased the expression of several genes [21,22]. Moreover, nicotine is known to induce the development of various cancers by causing DNA damage [23].

We also demonstrated that age-related changes in the expression of bitter taste receptors in smokers were significantly different from those in non-smokers, which is indicative of the potential accumulative effects of smoking. Although the association between age and the expression of bitter taste receptors is controversial and the relationship may not be consistent [24], it was reported to be greatly influenced by various environmental factors [25]. For instance, we previously reported that bitter taste receptor expression was influenced by exposure to a wide variety of foods; in other words the more food types people experience, the greater their expression of bitter taste receptors (presented at 15^th^ International Symposium on Olfaction and Taste at San Francisco). In the present study, we only included participants under 65 years of age. Thus, although additional factors might affect the relationship between age and bitter taste receptor expression, our results suggests that cigarette smoking impairs the acquisition of bitter taste receptors that occurs with aging and exposure to more tastes.

We observed no significant association between bitter taste receptor expression and smoking history, such as its duration and the number of cigarettes smoked daily. A possible explanation for this is that the expression of bitter taste receptors is so sensitive to environmental factors that it is conceivable that the expression would decrease to zero very quickly [25].

The current investigation has a number of methodological limitations. First, although we enlisted an age- and gender-matched non-smoker comparison group, several potential confounding factors were not considered that may affect TAS2R expression, such as genetic variability. Second, although this control group had no history of smoking, the potential effect of secondhand smoke was not investigated. However, unlike smoking, secondhand smoke does not enter the oral cavity, so its effect on TAS2R expression is potentially limited. Moreover, we mainly recruited adult males as the control group, and the Japanese Ministry of Health, Labour and Welfare reported that the prevalence of smoking among females is very low if their husbands do not smoke. Thus, although this should have been confirmed directly by questionnaire, the likelihood of exposure to secondhand smoke in our control group was low. Third, we did not investigate the mechanism by which cigarette smoke affects TAS2R expression, but it is assumed that there is a biochemical pathway that nicotine interferes with that decreases TAS2R expression. Fourth, although we have collected samples from the foliate papillae of the lateral portion of the tongue which is assumed to express bitter taste receptor well, and adopted standard method of the RT-PCR, no controls for presence of taste bud material for the RT-PCR experiments were performed. As it is state-of-the-art to perform and show control reaction such as samples amplified from not incubated with reverse transcription, further studies that adopted such method to improve the quality and reliability of the data are expected. Fifth, although we have shown that the lower phase of the TRIzol Reagent treated specimen included only genomic DNA fragments whose sizes are less than 200bp, suggesting that genomic DNA was fragmented into small factions by a process of homogenate we have not conducted enzymatic treatment of DNA degrading. Thus, there still remains possibility that the result of the present study is influenced by genomic DNA contamination. Sixth, although experimenter tried to obtain samples from tongue in the same manner in every participant, it may be rational criticism that the tongues of smokers and non-smokers are different in tissue. Concretely, it may be possible that the epithelium may be more keratinized in smokers than non-smokers, provoking the possibility that the difference in TAS2R expression between smokers and non-smokers derives from the difference in degree of keratinization rather than expression of the receptors. Similarly, the relation between smoking history and number of TAS2R expression may possibly reflect the effect of cigarette on lingual epithelium over years, rather than the mechanism we have assumed in the section of discussion. Further, although there was aging effect of number of expression of TAS2R among non-smoker between age of 20 and 65, it should be noted that such kind of aging effect may not continue after age of 65.

There remain some unanswered questions. For example, we were unable to examine the temporal relationship between smoking and TAS2R expression. It would be interesting to determine which receptor decreases its expression after the beginning of smoking. Additionally, it is not clear whether TAS2R expression recovers after smoking abstinence. Future research should investigate these areas.

## Conclusions

The current study demonstrated a significantly lower expression of the bitter taste receptor gene TAS2R in individuals who smoke cigarettes compared with non-smokers. Further, cigarette smoking may impede the acquisition of bitter taste receptors that occurs with experience.

## Abbreviations

GPCR: G-protein-coupled receptor; RT-PCR: Reverse transcriptase-polymerase chain reaction; TAS2R: Taste 2 receptor; TRC: Taste receptor cells.

## Competing interests

The authors declare that they have no competing interests.

## Authors’ contribution

MA designed the study. TT and TK performed experiments. MA recruited participants. MA analyzed data and wrote the paper. TK and NS also took part in writing the paper. All authors read and approved the final manuscript.

## References

[B1] Spangler JG Smoking and hormone-related disorders Prim Care 1999 26 499 511 10.1016/S0095-4543(05)70114-7 10436284

[B2] Cao S Yin X Wang Y Zhou H Song F Lu Z Smoking and risk of erectile dysfunction: systematic review of observational studies with meta-analysis PLoS One 2013 8 e60443 10.1371/journal.pone.0060443 23573257 PMC3616119

[B3] Spechler SJ Barrett esophagus and risk of esophageal cancer: a clinical review JAMA 2013 310 627 636 10.1001/jama.2013.226450 23942681

[B4] Scott DL Wolfe F Huizinga TWJ Rheumatoid arthritis Lancet 2010 376 1094 1108 10.1016/S0140-6736(10)60826-4 20870100

[B5] Gardener S Gu Y Rainey-Smith SR Keogh JB Clifton PM Mathieson SL Taddei K Mondal A Ward VK Scarmeas N Barnes M Ellis KA Head R Masters CL Ames D Macaulay SL Rowe CC Szoeke C Martins RN AIBL Research Group Adherence to a Mediterranean diet and Alzheimer's disease risk in an Australian population Transl Psychiatry 2012 2 e164 10.1038/tp.2012.91 23032941 PMC3565821

[B6] Xue WQ Qin HD Ruan HL Shugart YY Jia WH Quantitative association of tobacco smoking with the risk of nasopharyngeal carcinoma: a comprehensive meta-analysis of studies conducted between 1979 and 2011 Am J Epidemiol 2013 178 325 338 10.1093/aje/kws479 23785114 PMC3727336

[B7] Fowkes F Rudan D Rudan I Aboyans V Denenberg J McDermott M Norman P Sampson U Williams L Mensah G Criqui M Comparison of global estimates of prevalence and risk factors for peripheral artery disease in 2000 and 2010: a systematic review and analysis Lancet 2013 382 1329 1340 10.1016/S0140-6736(13)61249-0 23915883

[B8] Gromysz-Kałkowska K Wójcik K Szubartowska E Unkiewicz-Winiarczyk A Taste perception of cigarette smokers Ann Univ Mariae Curie Sklodowska Med 2002 57 143 154 12898832

[B9] Raffaelli R Baldinetti A Somma F Rumi G Tiberi F Variations in the taste function of smokers Minerva Somatol 1989 38 1253 1256 2628719

[B10] Szejtli J Szente L Elimination of bitter, disgusting tastes of drugs and foods by cyclodextrins Eur J Pharm Biopharm 2005 61 115 125 10.1016/j.ejpb.2005.05.006 16185857

[B11] Mela D Gustatory function and dietary habits in users and nonusers of smokeless tobacco Am J CLin Nutr 1989 49 482 489 2923081 10.1093/ajcn/49.3.482

[B12] Enoch M Harris C Goldman D Does a reduced sensitivity to bitter taste increase the risk of becoming nicotine addicted? Addict Behav 2001 25 399 404 11436931 10.1016/s0306-4603(00)00117-9

[B13] Cannon D Baker T Piper M Scholand M Lawrence D Drayna D McMahon W Villegas G Caton T Coon H MF L Associations between phenylthiocarbamide gene polymorphisms and cigarette smoking Nicotine Tob Res 2005 7 853 858 10.1080/14622200500330209 16298720

[B14] Snedecor S Pomerleau C Mehringer A Ninowski R Pomerleau O Differences in smoking-related variables based on phenylthiocarbamide “taster” status Addict Behav 2006 31 2309 2312 10.1016/j.addbeh.2006.02.016 16580152

[B15] Maehashi K Huang L Bitter peptides and bitter taste receptors Cell Mol Life Sci 2009 66 1661 1671 10.1007/s00018-009-8755-9 19153652 PMC11115905

[B16] Brockhoff A Behrens M Niv MY Meyerhof W Structural requirements of bitter taste receptor activation Proc Natl Acad Sci U S A 2010 107 11110 5 10.1073/pnas.0913862107 20534469 PMC2890741

[B17] Keller M Liu X Wohland T Rohde K Gast M Stumvoll M Kovacs P Tönjes A Böttcher Y TAS2R38 and its influence on smoking behavior and glucose homeostasis in the German Sorbs PLoS One 2013 8 e80512 10.1371/journal.pone.0080512 24312479 PMC3846558

[B18] Sekine H Takao K Yoshinaga K Kokubun S Ikeda M Effects of zinc deficiency and supplementation on gene expression of bitter taste receptors (TAS2Rs) on the tongue in rats Laryngoscope 2012 122 2411 2417 10.1002/lary.23378 23070743

[B19] Meng YG Liang J Wong WL Chisholm V Green fluorescent protein as a second selectable marker for selection of high producing clones from transfected CHO cells Gene 2000 242 201 7 10.1016/S0378-1119(99)00524-7 10721713

[B20] Tomassini S Cuoghi V Catalani E Casini G Bigiani A Long-term effects of nicotine on rat fungiform taste buds Neuroscience 2007 147 803 810 10.1016/j.neuroscience.2007.04.053 17560039

[B21] Satta R Maloku E Zhubi A Pibiri F Hajos M Costa E Guidotti A Nicotine decreases DNA methyltransferase 1 expression and glutamic acid decarboxylase 67 promoter methylation in GABAergic interneurons Proc Natl Acad Sci U S A 2008 105 16356 16361 10.1073/pnas.0808699105 18852456 PMC2570996

[B22] Shaykhiev R Sackrowitz R Fukui T Zuo WL Chao IW Strulovici-Barel Y Downey RJ Crystal RG Smoking-induced CXCL14 expression in the human airway epithelium links chronic obstructive pulmonary disease to lung cancer Am J Respir Cell Mol Biol 2013 49 418 425 10.1165/rcmb.2012-0396OC 23597004 PMC3824052

[B23] Hecht S Cigarette smoking and lung cancer: chemical mechanisms and approaches to prevention Lancet Oncol 2002 3 461 469 10.1016/S1470-2045(02)00815-X 12147432

[B24] Winkler S Garg A Mekayarajjananonth T Bakaeen L Khan E Depressed taste and smell in geriatric patients J Am Dent Assoc 1999 130 1759 1765 10.14219/jada.archive.1999.0133 10599179

[B25] Navarro-Allende A Khataan N El-Sohemy A Impact of genetic and environmental determinants of taste with food preferences in older adults J Nutr Elder 2008 27 267 276 10.1080/01639360802261920 19042575

